# Observations Regarding Chronic Heart Disease Complicating Pregnancy and Labour

**Published:** 1894-06-09

**Authors:** J. C. Webster

**Affiliations:** Assistant to the Professor of Midwifery in the University of Edinburgh


### OBSERVATIONS REGARDING CHRONIC HEART DISEASE COMPLICATING PREGNANCY AND LABOUR.

By J. C. Webster, M.D., F.R.C.P.Ed., Assistant to the Professor of Midwifery in the University of Edinburgh.

Notwithstanding the very serious nature of cardiac disease complicating pregnancy, labour, and the puerperium, it is only within recent years that this important subject has received any attention whatever in obstetrical text-books. Though a good deal has been done of late to point out the special dangers associated with the different cardiac affections, there is at the present time considerable difference of opinion both in regard to the causes of these dangers and the methods of dealing with them.

Before 1871, in most text-books—*e.g.*, Burns,[1] Davis,[2] Nägele's,[3] &c.—no mention whatever is made of heart diseases in connection with the pathology of pregnancy. There are various references to œdema, dyspnœa, cough, &c., but these are only considered symptomatically. Plenk [4] in his work refers to asthmatic and other conditions which disturb the breathing and so interfere with bearing-down efforts in labour. Ramsbotham [5] long ago described a case of mitral stenosis which died immediately after labour, the lungs being greatly congested. Busch[6] makes reference to heart disease merely as one of the conditions causing asthma which may be found as a complication of pregnancy. Hecker[7] in 1860 described a case of mitral stenosis which died during a premature labour and another of mitral incompetence which died on the twenty-third day after labour. In discussing the pathology, he thought that the lungs being congested from the heart condition were also much affected by diminution of the chest space by the pressure of the gravid uterus and so ceased action; he also believed that the heart might be exhausted as well from the effort of the labour. Cazeaux [8] in 1867 pointed out the importance of endocarditis as a cause of sudden death in labour, and he indicated, as regards treatment, that the second stage should be made as short as possible, artificial delivery being employed if necessary. Spiegelberg was the first, however, to give this subject special consideration, and his paper,[9] published in 1871, attracted a good deal of attention. He discussed the changes in the circulatory system resulting from pregnancy, comparing them with those brought about through the emptying of the uterus; he also considered the differences between mitral and aortic affections. In 1872 Peter,[10] of Paris, wrote an important paper, referring especially to the pulmonary dangers resulting from cardiac disease in pregnancy. In the same year Lebert[11] wrote an exhaustive article, in which we find some reference to the great dangers of affections of the right side of the heart. In 1875 Fritsch[12] discussed the subject, attacking especially some of Spiegelberg's views. In 1876 Lahs[13] sharply criticised Fritsch's opinions, and Fritsch replied in a valuable paper,[14] giving an account of several interesting cases. In 1876 Löhlein[15] discussed Spiegelberg's and Fritsch's views, and published some new observations of his own. In 1878 Angus Macdonald wrote his valuable work[16], containing a careful *resumé* of collected cases. In 1880 Porak[17] published a larger number of cases. In 1888 Berry Hart wrote his paper[18], dwelling mainly on the relation of mitral stenosis to the third stage. During the past twelve months interesting articles have been written by Leyden[19] and Sears.[20] Other papers have been published, but they need not be especially referred to.

It is very difficult to estimate accurately the mortality in cases of pregnancy and labour complicated by cardiac disease in which no treatment is carried out. The heaviest death-rate is in cases where pregnancy has occurred while the heart affection is of recent origin. Such cases are not common, however, and no satisfactory statistics can be given regarding them. In this paper we shall mainly devote our attention to those cases in which the heart lesion is in a chronic condition.

As is well-known, many of these may pass through pregnancy and labour perfectly safely and with little or no disturbance. The published mortality statistics are only approximate, and really refer to cases in which the symptoms were bad enough to attract the physician's attention. In 92 cases collected by Porak[21], the death-rate was 38·09 per cent.; in 118 by Remy[22], 33·8; in 20 by Leyden[23], 55; in 30 by Sears[24], 33·3; in 77 by Wessner[25], 37; in 58 by P. Müller[26], 39·65. When we analyse statistics more closely in order to determine the relative frequency and danger of the different varieties of heart lesion, we get the following results. (Right-sided affections are so rare that they can be put out of count. Our attention is therefore limited to mitral and aortic disease.)

*Mitral Stenosis.*—In Porak's 92 cases there were 13 of this variety, the mortality being 61·53 per cent.; in Macdonald's 31 cases, 14, the mortality being 64·4 per cent.; in Remy's 118 cases, 19, the mortality being 53·15 per cent.; in Sears' 30 cases, 14, the mortality 46·6 per cent.

*Mitral Regurgitation.*—In Porak's 92 cases there were 22 of this variety, the mortality being 13·63 per cent.; in Macdonald's 31 cases, 8, the mortality being 50 per cent.; in Remy's 118 cases, 29, the mortality being 20·68 per cent.

*Aortic Lesions.*—In Porak's 92 cases there were 13 with aortic lesions, the mortality being 23·07 per cent.; in Remy's 118 cases, 17, the mortality being 11·76 per cent.; in Sears' 30 cases, 6, the mortality being 16·66 per cent.

*Combined Mitral Lesions.*—In Porak's 92 cases there were 22 of this variety, the mortality being 45·45 per cent.; in Remy's 118 cases, 15, the mortality being 40 per cent.

*Complex Lesions (Mitral and Aortic).*—In Porak's 92 cases there were 22 of this variety, the mortality being 50 per cent.; in Remy's 118 cases, 16, the mortality being 18·75 per cent.; in Sears' 30 cases, 7, the mortality being 57·14 per cent.

As far, then, as we can judge from statistics there seems to be no doubt that the most fatal lesion is *Mitral Stenosis.*

When a patient suffering from a chronic cardiac valvular affection becomes pregnant what is likely to be the course of her history? The chief normal changes in the circulatory system during pregnancy are as follows: The vascular area is increased owing to the enormous development of vessels in the uterus and to the dilatation which occurs in the pelvic vessels, especially in the veins of the connective tissue, *e.g.*, para-metric, para-vaginal, and para-vesical. (This is well shown in sections[27] of the pregnant woman.)

The total quantity of blood in the maternal system is increased, there being in early pregnancy relative increase of the water and diminution of the red corpuscles, hæmoglobin, and albumen, while in the late months, according to Schrieder, Fehling, and others an augmentation of these latter elements[28] occurs. The increased work demanded of the heart is met by hypertrophy mainly of the left ventricle. There is still a dispute as to how much of the organ hypertrophies, and as to whether some dilatation occurs as well. Angus Macdonald's opinion is probably correct, viz., that all the chambers of the heart during the latter months of pregnancy become somewhat dilated. Undoubtedly increased work is demanded of the right as well as of the left side of the heart, because, as Charpentier so strongly insists, there is increased engorgement and tension in the pulmonary as well as in the systemic vascular system. There is also some increase in the frequency of the cardiac contractions during pregnancy.

There is still a dispute[29] regarding the interference with the thorax caused by the uterine swelling. According to Wintrich, Fabius, and others the vital capacity of the lungs is constant throughout pregnancy; according to Dohrn it is diminished. According to frozen sections, there is found in late pregnancy increased breadth and diminished depth in the thorax, but the vital capacity of the lungs is probably not much altered.

What new conditions are introduced when a valve of the heart is diseased? Great variations are found, depending upon such factors as the situation and extent of the lesion, the amount of compensation existing at the beginning of pregnancy, the general health of the patient, her habits, duties, &c. The deterioration of blood resulting from the pregnancy interferes somewhat with the cardiac nutrition. When the disease is recent there is always a danger of increase in the valvular vegetations that may be present owing to the increased fibrin-forming tendency in the blood. The main element of danger, however, is the increased work required from the heart as a result of the increased vascular area in the pregnant condition. The disturbances are so great that effects may be brought about which, in the non-pregnant state, might not be met with until after a course of years. The patient with heart disease, *cæteris paribus*, has a shorter life-expectation if she become pregnant, and her dangers increase with succeeding pregnancies.

In some cases, owing to the perfect response of the heart to its two-fold requirements, the patient may pass through her period of pregnancy with no more discomfort than may be found in normal cases. When, however, the cardiac compensation is not sufficient, one or more of the well-known symptoms, *e.g.*, pulmonary congestion and œdema, dyspnœa, cough, &c., make their appearance. In aortic disease, disturbances are much less common and less severe than in the case of mitral disease, and they appear in most cases during the late months of pregnancy. The symptoms are mainly palpitation and dyspnœa. In bad cases premature emptying of the uterus may result. It is in mitral disease that the most intense symptoms occur, and they may develop in the early, as well as in the late, months, and uterine action is apt to be set up, leading to the expulsion of the fœtus. The health of the fœtus tends to be impaired, both from the imperfectly oxygenated condition of the maternal blood as well as from the destruction of portions of the placenta by hæmorrhages into it from the maternal vessels. The increased risk in mitral cases, especially where stenosis exists, is the pulmonary congestion and dilatation and weakening of the right side of the heart. Sometimes the pregnant woman may die from the heart disease without emptying of the uterus occurring, but most cases of death before full-time has been reached occur in connection with or after a premature delivery.

Most cases which go on to full time become worse during the last few weeks. This is apt to be markedly the case if there is much dyspepsia, the flatulence combined with the increased size of the uterus exercising undue pressure on the diaphragm. Besides, patients often become despondent and nervous, lose their appetite, and suffer from sleeplessness at this time.

[1] The Principles of Midwifery, London, 1837. [2] Elements of Obst. Med., London, 2nd Ed., 1841. [3] Lehrbuch d. Geburtshülfe, Mainz. 5te. Aufl., 1852. [4] Anfangsgründe d. Geburtshülfe, Wien., 1795. [5] Pract. Observations on Mid., London, 1832. [6] Lehrbuch d. Geburtskünde, Berlin, 1842. [7] Hecker und Buhl; Klinik d. Geburtskünde, Leipsig, 1841. [8] Traite de l'Art des Accouchements, &c., Paris, 1867. [9] Archiv. f. Gyn., Bd. II., H ft. II., S. 236. [10] L'Union Médicale, Paris, Mars. 1872. [11] Archiv. f. Gyn., Bd. III., S. 38. [12] Archiv. f. Gyn., Bd. VIII., S. 373. [13] Archiv. f. Gyn., Bd. IX., S. 306. [14] Archiv. f. Gyn., Bd. X., S. 270. [15] Ueber das Verhalten des Herzens, Stuttgart, 1876. [16] The Bearing of Chronic Disease of the Heart upon Pregnancy, Parturition and Childbed, London, 1878. [17] De L'infl. Rècip. de la Grossesse et des Mal. de Cœur, Paris, 1880. [18] Obst. Trans., Edin., Vol. XIII. [19] Zeitschrift f. Klin. Med., Bd. XXIII. [20] Boston Med. and Surg. Journ., March 15, 1894. [21] Op. cit. [22] Thèse de Nancy, 1880. [23] Op. cit. [24] Op. cit. [25] Chron. Herzkrank und Puerperium, Bern., 1884. [26] Handbuch d. Geburtshülfe, Stuttgart, 1889, Bd. II., S. 908. [27] Barbour and Webster, Lab. Reports, Vol. II. [28] Arch. f. Gyn., Bd. XXXIX, S. 306. [29] Monatsch. f. Geburtskünde, Bd. XXIV., S. 414; Monatsch. f. Geburtskünde, Bd. XXVIII., S. 457, Spiegelberg, Lehrbuch d. Geburtshülfe, 1877, p. 63.

*(To be continued.)*

